# Mechanisms of beneficial effects of DPP-4 inhibitors as a promising perspective for the prevention/treatment of the disruption of cardio-cerebrovascular homeostasis

**DOI:** 10.3389/fphar.2025.1642333

**Published:** 2025-07-23

**Authors:** Katarina Tomović Pavlović, Marko Anderluh, Andrija Šmelcerović

**Affiliations:** ^1^Department of Pharmacy, Faculty of Medicine, University of Niš, Niš, Serbia; ^2^Department of Pharmaceutical Chemistry, Faculty of Pharmacy, University of Ljubljana, Ljubljana, Slovenia; ^3^Department of Chemistry, Faculty of Medicine, University of Niš, Niš, Serbia

**Keywords:** dipeptidyl peptidase-4, DPP-4 inhibition, cardio-cerebrovascular disorders, molecular mechanisms, therapeutic strategies

## Abstract

Cardio-cerebrovascular diseases are increasingly prevalent worldwide, with pathological changes in the heart and brain reinforcing each other. Diabetes is a major driver of comorbidity between these two systems and contributes to poor clinical outcomes. This review summarizes current evidence on shared risk factors and underlying mechanisms, with a particular focus on the role of dipeptidyl peptidase-4 (DPP-4) inhibitors as a potential therapeutic strategy for preserving cardio-cerebrovascular homeostasis. Growing evidence suggests that DPP-4 inhibitors offer benefits beyond glycemic control. These include improvements in endothelial function, reduction of oxidative stress and inflammatory responses, modulation of lipid and glucose metabolism, and regulation of blood pressure. Together, these actions support the anti-atherosclerotic and anti-thromboembolic properties of this drug class. These effects occur through both indirect pathways, via improved glycemic control, and direct cellular and molecular mechanisms. Although DPP-4 inhibitors are commonly used as second- or third-line agents in combination with other antidiabetic drugs, they have distinct advantages in specific populations. Notably, they are among the few hypoglycemic agents that are safe and effective in patients with impaired renal function—where the use of agents like metformin and SGLT-2 inhibitors is often contraindicated. In addition, DPP-4 inhibitors have shown favorable outcomes in elderly patients, particularly those aged 65 years and older. From a cardiovascular perspective, DPP-4 inhibitors have demonstrated protective effects against ischemic stroke, improved neurovascular function, and a reduction in major adverse cardiovascular events (MACEs). Importantly, they do not increase the risk of heart failure, unlike some other antidiabetic medications. While most cardiovascular outcome trials (CVOTs) involving DPP-4 inhibitors have shown neutral results, these studies were primarily designed to establish safety rather than demonstrate cardiovascular superiority. In conclusion, the pleiotropic effects, favorable safety profile, and suitability for vulnerable populations position DPP-4 inhibitors as promising agents in the management of cardio-cerebrovascular complications in diabetes. Further long-term, controlled clinical studies are warranted to fully establish their therapeutic potential across broader indications.

## 1 Introduction

The heart and brain are the cores of the human organism. Their pathological changes are mutually reinforcing and make them the leading causes of morbidity and mortality in both developing and developed countries (Liao et al., 2023). The burden of cardio-cerebrovascular diseases is increasing worldwide, which has become the greatest threat to human health and a major public health problem (Li et al., 2022). Cardiovascular (myocardial infarction, heart failure, and atrial fibrillation) and cerebrovascular (ischemic stroke, intracerebral and subarachnoid hemorrhage) diseases are often associated with common risk factors, such as diabetes mellitus, dyslipidemia, and hypertension and underlying pathologies like atherosclerosis and thromboembolism. Cardiovascular disorders cause the injury of cerebrovascular homeostasis, and *vice versa* (Nakai et al., 2022; Liao et al., 2023; Wichaiyo et al., 2024). For instance, cardiac arrhythmias, such as atrial fibrillation, predispose to cerebrovascular events, when blood clots in the atria and ventricles embolize the brain. In addition, ischemic stroke is a rare, but most-feared complication of myocardial infarction. All mentioned cardio-cerebrovascular disorders have complex etiopathogenesis and require a multidisciplinary diagnostic and therapeutic approach (Nakai et al., 2022). Diabetes increases the vulnerability of both, the brain and the heart in the pre-stroke stage. It establishes a suitable environment for ischemic stroke, drives systemic inflammation and trigger oxidative stress which exacerbate cardiac damage and induce cerebral-cardiac syndrome. Diabetes increases the incidence of cardiac complications (myocardial infarction, congestive heart failure, arrhythmia, cardiac arrest) after ischemic stroke, exacerbates mortality and worsens prognosis in the post-stroke state (Lin et al., 2021).

Endothelial dysfunction is a hallmark of cardio-cerebrovascular disorders and is associated with inflammation, vasoconstriction, and thrombosis. Dipeptidyl peptidase-4 (DPP-4) inhibition can maintain proper cardio-cerebrovascular system functioning by protecting the role of endothelial cells (Liao et al., 2023). It has been evidenced that inhibition of DPP-4 is associated with improved endothelial function, suppressed inflammation and oxidative stress, and regulated glycemia, lipidemia, and blood pressure (Chen et al., 2020). On the one hand, it is true that DPP-4 inhibition is involved in controlling cardiovascular risk factors by improving blood glucose control. On the other hand, additionally, DPP-4 inhibitors directly regulate the occurrence and progression of cardiovascular disorders through a variety of mechanisms (Chen et al., 2022). Glycemic control *per se* seems to fail in preventing the progression of diabetic cardiovascular complications. DPP-4 has the capability to inactivate not only incretins, but also a series of cytokines, chemokines, and neuropeptides involved in inflammation, immunity, and vascular function (Xie et al., 2018). Recently, we suggested that the administration of DPP-4 inhibitors may be beneficial in myocardial repair following infarction by the preservation of stem cell chemoattractant stromal cell-derived factor-1 (SDF-1) (Anderluh et al., 2016). Further, we offered an evidence-supported hypothesis that DPP-4 inhibitors might prevent fibrosis and suppress the entry to the irreversible phase of vascular remodeling in pulmonary hypertension (Anderluh et al., 2019). A significant plausible mechanism of protective effects of these possible multitarget agents in cardiovascular and renal pathology is anti-inflammatory activity (Tomovic et al., 2019). Here, the aim is to summarize the available evidence of potential mechanisms in the suppression of common risk factors and improvement of underlying pathology, to provide a rational scientific basis for the application of DPP-4 inhibitors in the prevention and treatment of cardio-cerebrovascular disorders.

## 2 Beneficial effects of DPP-4 inhibition on cardio-cerebrovascular diseasesʹ risk factors

Diabetes mellitus is often present long before a stroke occurs, causing inflammation, and disturbing the proper neurovascular functioning (Zhang et al., 2024). The blood-brain barrier might be harmed in a diabetes-induced inflammatory environment under the influence of elevated interleukin (IL)-1β, IL-6, tumor necrosis factor (TNF)-α levels, and advanced glycation end products (AGEs) accumulation (Yang et al., 2024). DPP-4 inhibitors have a beneficial impact on improving impaired cerebrovascular structure and function (Cao et al., 2021). Inhibition of DPP-4 protects brain endothelial cells under hyperglycemic and hypoxic conditions by the suppression of TNF-α-induced nuclear factor (NF)-κB p65 accumulation and cytokine IL-6, IL-8, intercellular adhesion molecule-1 (ICAM-1), and vascular cell adhesion molecule-1 (VCAM-1) expression (Yang et al., 2024), suppressing advanced glycation end products and their receptors (AGE-RAGE) axis, and pro-fibrotic cytokines expression, as well (Chen et al., 2020; Cao et al., 2021).

Under the influence of risk factors such as elevated glycemia, blood pressure, and cholesterol levels, endothelial dysfunction happens, characterized by inflammation, oxidative stress, decreased nitric oxide (NO) biosynthesis, endothelial-mesenchymal transition, and cell senescence. Endothelial dysfunction is an early indicator of atherosclerosis, and aggravated by diabetes it is an essential factor in stroke development. DPP-4 inhibitors increase NO bioavailability and reduce endothelin-1-induced basilar arteries contraction, improving diabetic cerebrovascular dysfunction (Hardigan et al., 2016; Yang et al., 2024).

Lipid profile, triglycerides (TGs)/high-density lipoprotein (HDL) cholesterol ratio, is a predictor for early vascular aging. Postprandial hypertriglyceridemia contributes to endothelial dysfunction and accelerates the development of atherosclerosis (Cao et al., 2021). Besides imbalanced lipid metabolism, atherosclerosis is characterized by maladaptive inflammation caused by macrophage accumulation in the arterial wall. Expression of DPP-4 on monocytes/macrophages is increased in obese atherosclerotic patients and is positively correlated with the levels of triglycerides and non-HDL cholesterol (Rao et al., 2018). DPP-4 inhibitors decrease the level of low-density lipoprotein (LDL) cholesterol, triglycerides, total cholesterol, and free fatty acids, and increase the HDL level in human models (Cao et al., 2021). DPP-4 significantly contributes to the inflammation and insulin resistance in obesity. By improving adipose tissue inflammation, promoting its remodeling, and decreasing ectopic deposition, DPP-4 inhibitors regulate adipose tissue’s function in metabolism and lipid homeostasis (Guo et al., 2024). DPP-4 inhibitors affect the expression of liver enzymes responsible for lipid oxidation and biosynthesis by modulating the GLP-1 signaling pathway, leading to decreased intestinal lipid synthesis, secretion and absorption. They also increase plasma norepinephrine levels by activating the sympathetic nervous system, which in turn accelerates postprandial lipid mobilization (Chen et al., 2022).

Aging-related vascular wall functional and structural alterations contribute to the pathogenesis of hypertension, dyslipidemia, atherosclerosis, heart failure, cerebrovascular and neurodegenerative diseases. DPP-4 inhibitors play a protective role in reversing vascular aging through the promotion of endothelial cells proliferation and migration, alleviating senescence, by increasing SDF-1α, vascular endothelial growth factor (VEGF), superoxide dismutase, and reducing nicotinamide adenine dinucleotide phosphate (NADPH) oxidase expression, regulating AMP-activated protein kinase (AMPK)/sirtuin 1/nuclear factor erythroid 2-related factor 2 (Nrf2) signaling pathway, improving NO bioavailability, decreasing pro-apoptotic Bax and caspase 3, while promoting the expression of anti-apoptotic B-cell lymphoma 2 (Bcl-2) (Chen et al., 2020; Cao et al., 2021).

**FIGURE 1:**
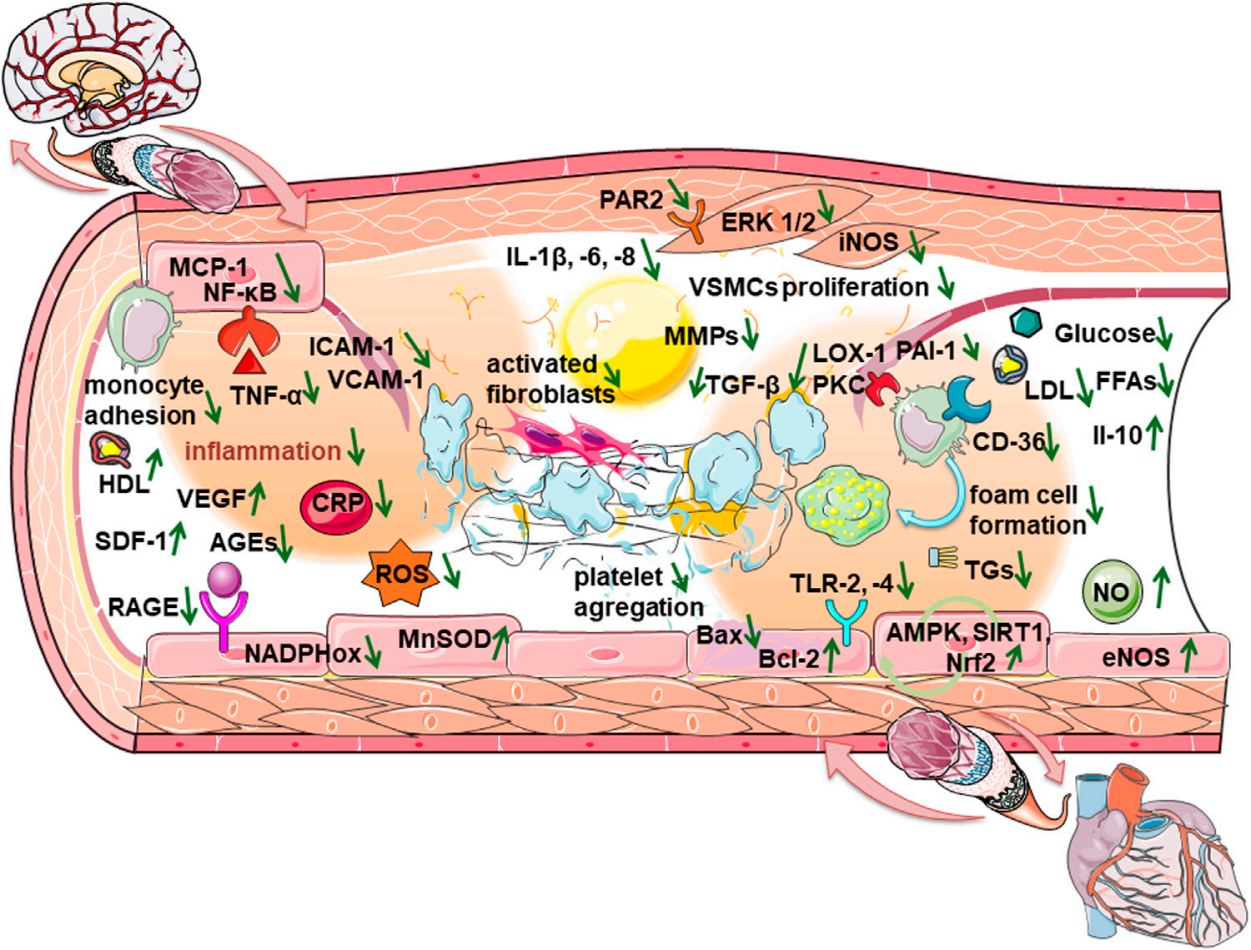
Mechanisms of beneficial effects of DPP-4 inhibitors when endothelial dysfunction progresses to disruption of cardio-cerebrovascular homeostasis.

Therefore, with the treatment of diabetes, which is their primary indication, DPP-4 inhibitors regulate glycemia, covering inflammation, endothelial dysfunction, oxidative stress, and dyslipidemia with favorable pleiotropic effects, and thus suppress the aforementioned risk factors, and prevent disruption of normal cardio-cerebrovascular functioning (Table 1; Figure 1).

**TABLE 1:** Effects of DPP-4 inhibitors associated with vascular protection and mechanisms evidenced in human cell lines.

Cells	DPP-4 inhibitor	Function	Mechanism	References
HUVECs	Saxagliptin	Amelioration of ox-LDL-induced endothelial dysfunction	Reduced production of TNF-α, IL-1β, VCAM-1, ICAM-1, suppressed adhesion of monocytes to endothelial cells, prevented decrease of NO generation, mitigated ROS production, suppressed expression of NADPH oxidase, and NF-κB activation, inhibitory effect on endothelial LOX-1 expression	Ma et al. (2019)
	Anagliptin	Amelioration of high glucose-induced endothelial dysfunction	Reduced ROS generation, NADPH oxidase, and IL-1β expression, prevented reduction of SIRT1	Jiang et al. (2019)
	Saxagliptin	Improvement of endothelial senescence	Upregulation of MnSOD, abolished improvement of NADPH oxidase activity, activation of signaling pathway AMPK/SIRT1/Nrf2	Chen et al. (2020)
	Teneligliptin	Amelioration of oxidative stress and apoptotic phenotype in overcoming the metabolic memory effect induced by chronic exposure to high glucose	Reduced ROS levels, increased expression of the anti-apoptotic Bcl2, decreased the pro-apoptotic Bax and caspase-3	Pujadas et al. (2017)
	Gemigliptin	Protection of the vascular endothelium against inflammatory diseases such as atherosclerosis	Increased levels of phosphorylated AMPK and Akt, decreased NF-κB levels, reduced LPS-induced expression of TNF-α, MCP-1, IL-1β, IL-6, and VCAM-1, suppressed foam cell formation from THP-1 macrophages	Hwang et al. (2015)
	Linagliptin	Effects on AGEs evoked endothelial cell damage	Inhibition of the AGE-induced ROS generation, RAGE, ICAM-1, and PAI-1 expression	Ishibashi et al. (2013)
	Sitagliptin	Prevention of autophagy in cells exposed to high-glucose	Prevention of the increase of pro-inflammatory IL-6, and IL-8	Chang et al. (2021)
HAECs	Trelagliptin	Inhibition of IL-1β-induced endothelial inflammation and monocyte attachment	Inhibited expression of MCP-1, IL-6, VCAM-1, and ICAM-1, suppression of NF-κB signaling pathway, and monocyte adhesion	Meng et al. (2020)
Primary human retina endothelial cells	Linagliptin	Vascular protection in retinal endothelial cells	Inhibited TNF-α-induced secretion of IL-6 and IL-8, suppressed expression of ICAM-1 and VCAM-1, and adhesion of monocytes to endothelial cells	Li et al. (2019)
Primary human coronary artery SMCs	K579	Suppression of para- or endocrine actions of soluble DPP-4 on the vascular wall	Suppressed proliferation of hVSMCs, decreased phosphorylation of NF-κB p-65, reduced IL-6, IL-8, and MCP-1	Wronkowitz et al. (2014)
Human lung fibroblasts	Sitagliptin	Promise for the treatment of fibroproliferative disorders	Alleviated extracellular matrix deposition by downregulating the TGF-β/Smad-3 pathway	Liu et al. (2020b)
THP-1 macrophages	Sitagliptin	Repression of foam cell formation following treatment of macrophages with ox-LDL	Decreased expression of CD36 and LOX-1 scavenger receptors, inhibited phospho-PKC and IL-6 expression	Dai et al. (2014a)
	Sitagliptin	Suppression of inflammatory state and immune response in atherosclerosis	Suppression of TLR-4 and IL-1β expression through PKC inhibition	Dai et al. (2014b)

AGE, advanced glycation end products; Akt, protein kinase B; AMPK, AMP-activated protein kinase; Bcl2, B-cell lymphoma 2; CD36, fatty acid translocase; HAECs, human aortic endothelial cells; HUVECs, human umbilical vein endothelial cells; hVSMC, human vascular smooth muscle cells; ICAM-1, intercellular adhesion molecule-1; IL-1β, interleukin-1β; LDL, low-density lipoprotein; LOX-1, oxidized LDL receptor 1; LPS, lipopolysaccharide; MCP-1, monocyte chemoattractant protein-1; MnSOD, manganese superoxide dismutase; NADPH oxidase, nicotinamide adenine dinucleotide phosphate oxidase; NF-κB, nuclear factor-κB; NO, nitric oxide; Nrf2, nuclear factor erythroid 2-related factor 2; PAI-1, plasminogen activator inhibitor-1; PKC, protein kinase C; RAGE, receptor for AGE; ROS, reactive oxygen species; SIRT1, sirtuin 1; TGF-β, transforming growth factor β; TLR4, Toll-like receptor 4; TNF-α, tumor necrosis factor-α; VCAM-1, vascular cell adhesion molecule-1.

## 3 Favorable effects of DPP-4 inhibition in common underlying pathology of cardio-cerebrovascular diseases

DPP-4 inhibitors show anti-atherosclerotic effects by reducing the number of monocytes, TNF-α-induced monocyte migration and infiltration. Inhibition of DPP-4 suppresses foam cell formation, by down-regulating protein kinase B (Akt)/AMPK-dependent NF-κB signaling, reducing the expression of scavenger receptors lectin-type oxidized LDL receptor 1 (LOX-1) and fatty acid translocase (CD36) on macrophages, suppressing Toll-like receptor 4 (TLR4) signaling, and reducing the release of IL-1β in human macrophages by decreasing protein kinase C activity (Liu H. et al., 2020). Soluble DPP-4 induces proliferation of human vascular smooth muscle cells (VSMCs) through the activation of extracellular signal-regulated kinase (ERK) 1/2, the mitogen-activated protein kinase (MAPK) signaling pathway. Additionally, soluble DPP-4 activates NF-κB resulting in elevated pro-inflammatory cytokines IL-6, IL-8, MCP-1, and inducible nitric oxide synthase (iNOS) expression in human VSMCs. Soluble DPP-4 might be an agonist for the protease-activated receptor (PAR) family, highly expressed in epithelial and vascular/nonvascular smooth muscle cells. PAR2 silencing prevents DPP-4-induced proliferation and inflammation (Wronkowitz et al., 2014). DPP-4 inhibitors suppress smooth muscle cell proliferation, migration and expression of the chemokines, such as monocyte chemoattractant protein-1 (MCP-1) and VCAM-1, activate the Nrf-2 signaling pathway and reduce the expression of pro-inflammatory IL-6, TNF-α, C-reactive protein (CRP), MCP-1, ICAM-1, and metalloproteinases (MMPs), increasing the levels of the anti-inflammatory IL-10. Additionally, it reduces the concentrations of MMP-2 and MMP-9 in the circulation, and MMP-9 expression in the plaque, while increasing collagen content that stabilizes the plaque (Liu H. et al., 2020; Cao et al., 2021).

Plasminogen activator inhibitor-1 (PAI-1) is an important regulator of embolism, and its elevated level is one of the biomarkers of thrombosis-related disorders. DPP-4 inhibitors downregulate PAI-1 levels in diabetic patients and animals, possibly by reducing PAI-1 synthesis in endothelial cells (Liu H. et al., 2020). Insulin resistance is associated with hypercoagulability, platelet hypersensitivity, endothelial dysfunction, and impaired fibrinolysis, resulting in high thromboembolic risk. Type 2 diabetes and atrial fibrillation share common thrombotic pathways. Diabetic hearts are characterized by high fibroblast activity, associated with oxidative stress and inflammation, aggravated by elevated CRP and TNF-α, leading to an increased incidence of atrial fibrillation. Oxidative stress upregulates transforming growth factor β (TGF-β) resulting in the activation of fibrotic signaling (Liu X. et al., 2020). In addition, the increased production of AGEs also contributes to atrial fibrosis through the upregulation of connective tissue growth factors (Leopoulou et al., 2023). DPP-4 inhibition contributes to atrial remodeling and improves mitochondrial function, thereby reducing the risk of atrial fibrillation which predisposes to cerebrovascular events (Chang et al., 2017; Nakai et al., 2022).

In short, when endothelial dysfunction progresses to atherosclerosis and the risk of developing thromboembolism increases, DPP-4 inhibitors show beneficial effects there (Table 1; Figure 1) by suppressing macrophage infiltration, the formation of foam cells, and the proliferation of smooth muscle cells. DPP-4 inhibitors also have anti-fibrotic effects, reduce the risk of atrial fibrillation, and consequently suppresses the disruption of cerebrovascular homeostasis.

## 4 Clinical evidence on the effects of DPP-4 inhibitors on cardio- and cerebrovascular events, challenges and considerations

In recent years, five clinical trials (EXAMINE, SAVOR-TIMI 53, TECOS, CARMELINA and CAROLINA) have been conducted on the effects of DPP-4 inhibitors (alogliptin, saxagliptin, sitagliptin, and linagliptin, respectively) on major adverse cardiovascular events (MACE) (cardiovascular death, non-fatal myocardial infarction, and non-fatal stroke). Each of these trials was intended mainly to demonstrate the cardiovascular safety of gliptins and showed that DPP-4 inhibitors did not increase the risk of MACE. EXAMINE and SAVOR-TIMI 53 trials demonstrated the cardiovascular safety of alogliptin and saxagliptin in patients with type 2 diabetes and acute coronary syndrome (Zannad et al., 2015; Scirica et al., 2013). Trial TECOS confirmed the neutral effect of sitagliptin on MACE and hospitalization for unstable angina (Cornel et al., 2016). The cardiovascular safety of linagliptin was confirmed by two trials: CARMELINA (McGuire et al., 2019) where linagliptin slowed down the progression of albuminuria in patients with type 2 diabetes and high cardiovascular and renal risk, and CAROLINA (Espeland et al., 2021), where linagliptin and glimepiride efficacy and safety was compared in patients with type 2 diabetes and shown that linagliptin had significantly lower risk of hypoglycemia and falls or fractures than glimepiride. We have to emphasize that in the SAVOR-TIMI 53 trial, saxagliptin use increased the risk of hospitalization for heart failure (HF) by 27%, particularly in the first year and especially in diabetic patients. This was not related to increased cardiovascular or all-cause mortality. Other DPP-4 inhibitors did not increase the risk of HF hospitalization. The duration of follow-up in the SAVOR-TIMI 53 trial may have been insufficient to fully assess the HF risk of DPP-4 inhibition and further, the safety signal could be a chance finding due to multiple testing. The heterogeneity of the comparator drugs and their HF risks must be considered as differences in the distribution of the use of non-study drugs (and their HF risks) may explain some heterogeneity in the risk estimates. In summary, in these trials, the patients were given a selected DPP-4 inhibitor or placebo based on standard diabetic and cardiovascular therapy to control the optimal level of glycemia, leading to small differences in hemoglobin A1c (HbA1c) between the groups, which may weaken the potential benefits of DPP-4 inhibitors via decreasing HbA1c (Filion and Suissa, 2016; Cao et al., 2021; Chen et al., 2022). The abovementioned cardiovascular outcome trials were primarily designed to prove safety by showing noninferiority versus placebo and not to demonstrate superiority of DPP-4 inhibitors. The duration of these trials was rather short (2–3 years), so that the difference in hyperglycemia exposure was probably too low to show any difference in cardiovascular outcomes, especially in diabetic patients with already advanced cardiovascular disorders (Scheen, 2018). Likewise, not all GLP-1 receptor agonists have demonstrated clear and comparable cardiovascular benefits, which may vary in association with the specific molecule and the current setting of application, studies designed to assess non-inferiority to placebo in terms of cardiovascular safety, and to assess superiority to placebo in terms of efficacy, in the follow-up duration variable, ranging from 16 months to 8.6 years trials (Gaggini et al., 2025). Despite neutral results of mentioned clinical trials, the effects of DPP-4 inhibitors associated with vascular protection, through the improvement of endothelial function, suppression of inflammation and oxidative stress, regulation of glycemia, lipidemia, and blood pressure, anti-atherosclerotic and anti-thromboembolic effects, mediated by GLP-1, or many other substrates/ligands of this widely distributed protease (catalytically active and with receptor-like function), depending on many factors such as certain type of cell, tissue, regional expression of cytokines, chemokines, other mediators, signaling cascades and entity of disorders, have been evidenced in human cell lines and subjects (Tables 1, 2). Therefore, we still believe that DPP-4 inhibitors might contribute to the suppression of the occurrence and progression of cardio-cerebrovascular disorders. Further evaluation in long-term controlled trials and clinical practice is still needed to strengthen support for pleiotropic effects and potential broadened indications of DPP-4 inhibitors.

**TABLE 2:** Effects of DPP-4 inhibitors associated with vascular protection and mechanisms evidenced in human subjects.

Patients	DPP-4 inhibitor	Function	Mechanism	References
Patients with type 2 diabetes	Sitagliptin	Important insights into therapeutic implications in diabetic-related atherosclerotic diseases	Decreased serum levels of CRP, serum and monocyte TNF-α, increased expression of IL-10 in peripheral blood monocytes, and serum	Satoh-Asahara et al. (2013)
	Sitagliptin	Potential favorable cardiovascular implications	Increased plasma concentrations of SDF-1α, and circulating endothelial progenitor cells, decrease of MCP-1	Fadini et al. (2010)
Obese patients with type 2 diabetes	Sitagliptin	Potential contribution of potent and rapid anti-inflammatory effect to the inhibition of atherosclerosis	Decreased expression of TNF-α, TLR-4, TLR-2, and NF-κB DNA binding in mononuclear cells, fall of plasma concentrations of CRP, IL-6, and free fatty acids	Makdissi et al. (2012)
Diabetic hypertensive patients	Vildagliptin	Efficient and safe treatment option against diabetes mellitus comorbid with hypertension	Reduced blood pressure, lower total cholesterol, triglycerides, and LDL, elevated HDL and serum VEGF levels	El-Naggar et al. (2019)
Patients with diabetes on hemodialysis	Teneligliptin	Anti-atherothrombotic effect in the prevention of cardiovascular disease in patients with diabetes on hemodialysis	Significantly reduced plasma levels of PDMPs, PAI-1, and sVCAM-1	Okuda et al. (2016)
Patients with type 2 diabetes	Linagliptin	Improvement of endothelial and neurovascular microvascular function	An increase in axon reflex-dependent vasodilation, decrease in concentrations of IFN-γ, IL-6, IL-12, and MIP-1α	Baltzis et al. (2016)

CRP, C-reactive protein; FFAs, free fatty acids; HDL, high-density lipoprotein; IFN-γ, interferon gamma; IL, interleukin; LDL, low-density lipoprotein; MCP-1, monocyte chemoattractant protein-1; MIP-1α, macrophage inflammatory protein-1α; NF-κB, nuclear factor-κB; PAI-1, plasminogen activator inhibitor-1; PDMPs, platelet-derived microparticles; SDF-1α, stromal cell-derived factor-1α; TLR4, Toll-like receptor 4; TNF-α, tumor necrosis factor-α; VCAM-1, vascular cell adhesion molecule-1; VEGF, vascular endothelial growth factor.

In the assessment of risks of cardiovascular diseases associated with antidiabetic drugs in patients with type 2 diabetes, Ou et al. found that DPP-4 inhibitor users had a significantly lower risk for MACE, stroke, myocardial infarction, and HF than that of meglitinides and insulin, but higher than that of metformin users (Ou et al., 2016). Despite their relatively high cost, DPP-4 inhibitors are frequently prescribed as a third-line add-on therapy for patients with type 2 diabetes who exhibit inadequate glycemic control on combination treatment with metformin and sulfonylureas. A recent real-world evidence suggests that DPP-4 inhibitors may offer more significant therapeutic advantages even when used earlier in the treatment algorithm. A large, nationwide cohort study involving 113,051 patients with diabetes demonstrated that the use of DPP-4 inhibitors as a second-line add-on to metformin was associated with significantly lower risks of stroke, major adverse cardiovascular events (MACEs), and all-cause mortality when compared to sulfonylurea-based regimens. Furthermore, when used as a third-line agent in combination with both metformin and sulfonylurea, DPP-4 inhibitors continued to show superior outcomes. Specifically, they were associated with a significantly reduced risk of stroke and all-cause mortality in comparison to acarbose, and showed even greater benefit over meglitinides, with reduced risks of stroke, heart failure (HF), and all-cause mortality. These findings suggest that DPP-4 inhibitors, whether employed as second- or third-line therapy, confer meaningful cardiovascular protection and contribute to a reduction in overall mortality. Importantly, they do so without increasing the risk of heart failure or hypoglycemia, which are notable concerns with some other antidiabetic drug classes such as acarbose, meglitinides, and thiazolidinediones (Ou et al., 2017). From a clinical perspective, this is particularly relevant given that a substantial proportion of patients with type 2 diabetes (especially those with advanced cardiovascular disease) also experience varying degrees of renal impairment. In such patients, the therapeutic landscape becomes more limited, as many hypoglycemic agents, including metformin and sodium-glucose cotransporter 2 (SGLT-2) inhibitors, may be contraindicated due to safety concerns. In contrast, DPP-4 inhibitors have been shown to be safe and well-tolerated even in the presence of compromised renal function, making them a valuable option for these high-risk patients (Scheen, 2018). Renal function should be assessed before initiating therapy with either GLP-1 receptor agonists or SGLT-2 inhibitors. Use of the GLP-1 receptor agonist exenatide has been associated with cases of acute renal insufficiency or worsening of chronic renal failure. Generally, use of SGLT-2 inhibitors in patients with low glomerular filtration rate is not recommended or contraindicated, depending on the particular agent. SGLT-2 inhibitors might be less efficacious and the risk of adverse effects such as diabetic ketoacidosis increased (Gurgle et al., 2016). For SGLT-2 inhibitors the dose adjustment recommendations should be referred to per the kidney functions, specific to the agent within the class, as well closely monitor for genital mycotic infections and volume depletion (Brown et al., 2021). Sitagliptin as a second-line oral hypoglycemic agent was associated with a lower risk of new-onset atrial fibrillation in patients with diabetes compared with other drugs as second hypoglycemic agents including sulfonylureas, α-glucosidase inhibitors, meglitinides, and thiazolidinediones (Chang et al., 2017). Sitagliptin was also shown to be associated with a lower risk of coronary heart disease and ischemic stroke in diabetic patients (Yang et al., 2016). Linagliptin was shown to be protective for both macro- and microvascular complications of diabetes in preclinical and clinical models. Namely, linagliptin treatment for 3 months improved endothelial function relative to voglibose, accompanied by amelioration of glycemic, renal, and cardiometabolic parameters, in patients with newly diagnosed type 2 diabetes and coronary artery disease (Aroor et al., 2018; Koyama et al., 2018). Some nationwide cohort studies in Taiwan have shown that DPP-4 inhibitors can reduce the incidence of new-onset atrial fibrillation in elderly patients (Chen et al., 2017). Among patients aged 65 years or older with type 2 diabetes, DPP-4 inhibitors were associated with a lower mortality rate, fewer MACE, and no increase in HF or hypoglycemia, which supports the concept that elderly individuals benefit from DPP-4 inhibitors (Shih et al., 2016). For GLP-1 receptor agonists, treatment deintensification of these agents, particularly in older and frail individuals (approximately ≥65 years), is recommended to avoid hypoglycaemia and hypovolaemia. Moreover, retinopathy screening should be done before initiation (for semaglutide only) (Brown et al., 2021).

Enzan et al. found that DPP-4 inhibitors use was associated with better long-term outcomes and a lower incidence of cardiovascular death or HF hospitalization in patients with heart failure with preserved ejection fraction, but not in patients with heart failure with mildly reduced or reduced ejection fraction, and diabetes (Enzan et al., 2023). In another randomized placebo-controlled trial in patients with type 2 diabetes and HF by McMurray et al., vildagliptin had no major effect on left ventricular ejection fraction but did lead to an increase in left ventricular volumes (McMurray et al., 2018). However, there was no other indication of worsening heart failure status, so more evidence is needed regarding the safety of DPP-4 inhibitors in patients with established heart failure and left ventricular systolic dysfunction. The role of GLP-1 receptor agonists among patients with HF also remains unclear, and their effects may differ in patients with and without established HF, particularly those with decompensated heart failure with reduced ejection fraction (Nassif and Kosiborod, 2019). Subcutaneous and long acting GLP-1 receptor agonists show cardioprotection, but evidence is inconclusive for short-acting and oral long-acting medications (Davies et al., 2022). Clinical evidence continues to underscore the potential of DPP-4 inhibitors in mitigating cerebrovascular risk among patients with type 2 diabetes. A case-control study conducted by Lai et al. (2017) demonstrated that sustained use of a DPP-4 inhibitor for more than 1 year was significantly associated with a reduced risk of ischemic cerebrovascular disease. Notably, the authors emphasized that the protective effect of DPP-4 inhibitors becomes particularly evident when the duration of use reaches or exceeds 1 year. This finding suggests that future clinical trials should be specifically designed to evaluate the long-term cerebrovascular benefits of DPP-4 inhibitors, rather than focusing solely on short-term glycemic outcomes.

In addition to their cerebrovascular effects, DPP-4 inhibitors have a well-established role in glycemic management. They are capable of reducing hemoglobin A1c (HbA1c) levels by approximately 0.5%–1%. Importantly, with continued use over a year or longer, there is evidence of a gradual and sustained reduction in HbA1c levels, contributing to improved long-term glycemic control. This progressive improvement is not only beneficial for metabolic stability but also appears to further lower the risk of ischemic cerebrovascular complications, as shown in studies such as that by Cheng et al. (2020). However, the interpretation of HbA1c trends should be made cautiously. As highlighted by Pei et al. (2023), the observed association between HbA1c variability and adverse cardiovascular or cerebrovascular events may not solely reflect the impact of glycemic fluctuations themselves. Rather, this variability may signal underlying differences in patients’ baseline characteristics, such as comorbidities, treatment adherence, or disease severity, which could confound the relationship between glycemic control and vascular outcomes.

Davies et al. concluded that DPP-4 inhibitors are more widely used than SGLT2 inhibitors or GLP-1 receptor agonists, despite the lack of evidence that DPP-4 inhibitors improve cardiorenal outcomes in patients with type 2 diabetes. (Davies et al., 2022). The relative benefits of these drugs in different populations remain undefined. The meta-analysis showed that both GLP-1 receptor agonists and SGLT-2 inhibitors reduce the risk of myocardial infarction and cardiovascular death in patients with known established atherosclerotic cardiovascular disease, whereas neither reduces the risk of MACE in patients with only multiple risk factors. Only GLP-1 receptor agonists reduce the risk of stroke, while SGLT-2 inhibitors reduce the relative risk of hospitalization for heart failure. The reduction in MACE may require more time to become evident in patients with lower risk (Brown et al., 2021; Gaggini et al., 2025; Zelniker et al., 2019).

Cardiovascular disease remains the prognosis-limiting factor, and the main source of comorbidity and mortality in type 1 diabetes. To date, no cardiovascular outcome trials have been published for type 1 diabetes as an adjuvant to standard insulin therapy. Some scientific arguments have been proposed for the positive effects of the agents typically used in type 2 diabetes treatments when applied in type 1 diabetes, in modifying cardiovascular risk factors as well. *In vitro*, human, and animal studies have suggested that DPP-4 inhibitors improve β-cell function, reduce postprandial glucagon secretion and attenuate autoimmunity in type 1 diabetes. SGLT-2 inhibitors also showed promising results in persons with type 1 diabetes, but with a risk of diabetic ketoacidosis. GLP-1 receptor agonists directly target β-cells to improve their function and also protect them from immune-mediated inflammatory stress. Referring to the high and growing prevalence of metabolic syndrome, and cardiovascular comorbidity in type 1 diabetes, these agents should be more extensively considered as being potentially disease-modifying drugs in the future and should be investigated for hard cardiovascular endpoints (Aberer et al., 2022; von Scholten et al., 2021).

Taken together, the growing body of evidence supports the hypothesis that DPP-4 inhibitors may offer potential benefits for the prevention and management of impaired cardio-cerebrovascular homeostasis. Their potential long-term benefits, particularly in reducing ischemic cerebrovascular events, merit further exploration through well-structured, long-duration clinical trials.

## 5 Conclusion

Diabetes mellitus plays a significant role in the interplay of cardio-cerebrovascular comorbidity, increasing the incidence and contributing to a poorer prognosis. Among the available glucose-lowering agents, dipeptidyl peptidase-4 (DPP-4) inhibitors have demonstrated certain advantages. These advantages are associated with a reduced occurrence and progression of cardiovascular disorders through various mechanisms. Furthermore, these agents have been found to be effective and safe when used in patients with impaired renal function. Additionally, they have been shown to be beneficial for individuals aged 65 years and older. The relatively brief clinical cardiovascular outcome trials (CVOTs) for DPP-4 inhibitors have been primarily designed to demonstrate safety, i.e., non-inferiority to placebo, rather than to establish clinical superiority. These trials have largely yielded neutral results, with limited differences in cumulative hyperglycemia exposure. These factors may have prevented the detection of cardiovascular superiority (Scheen, 2018). Conversely, a mounting body of experimental evidence from human cell lines and clinical trials indicates that DPP-4 inhibitors may confer vascular protection, enhance endothelial function, and suppress inflammation and oxidative stress. These findings suggest a potential for DPP-4 inhibitors to regulate glycemia, lipidemia, and blood pressure, along with anti-atherosclerotic and anti-thromboembolic properties. The hypothesis suggests that these inhibitors may contribute to the prevention and attenuation of impaired cardio-cerebrovascular homeostasis. In order to provide comprehensive validation of their pleiotropic effects and to support a broader range of clinical indications, further long-term controlled studies and real-world clinical evaluations are necessary. The results of these additional studies are eagerly awaited.

## References

[B1] Aberer F. Pieber T. R. Eckstein M. L. Sourij H. Moser O. (2022). Glucose-lowering therapy beyond insulin in type 1 diabetes: a narrative review on existing evidence from randomized controlled trials and clinical perspective. Pharmaceutics 14, 1180. 10.3390/pharmaceutics14061180 35745754 PMC9229408

[B2] Anderluh M. Kocic G. Tomovic K. Kocic H. Smelcerovic A. (2019). DPP-4 inhibition: а novel therapeutic approach to the treatment of pulmonary hypertension? Pharmacol. Ther. 201, 1–7. 10.1016/j.pharmthera.2019.05.007 31095977

[B3] Anderluh M. Kocic G. Tomovic K. Kocic R. Deljanin-Ilic M. Smelcerovic A. (2016). Cross-talk between the dipeptidyl peptidase-4 and stromal cell-derived factor-1 in stem cell homing and myocardial repair: potential impact of dipeptidyl peptidase-4 inhibitors. Pharmacol. Ther. 167, 100–107. 10.1016/j.pharmthera.2016.07.009 27484974

[B4] Aroor A. R. Manrique-Acevedo C. DeMarco V. G. (2018). The role of dipeptidylpeptidase-4 inhibitors in management of cardiovascular disease in diabetes; focus on linagliptin. Cardiovasc. Diabetol. 17, 59–15. 10.1186/s12933-018-0704-1 29669555 PMC5907287

[B5] Baltzis D. Dushay J. R. Loader J. Wu J. Greenman R. L. Roustit M. (2016). Effect of linagliptin on vascular function: a randomized, placebo-controlled study. J. Clin. Endocrinol. Metab. 101, 4205–4213. 10.1210/jc.2016-2655 27583476 PMC5095255

[B6] Brown E. Heerspink H. J. Cuthbertson D. J. Wilding J. P. (2021). SGLT2 inhibitors and GLP-1 receptor agonists: established and emerging indications. Lancet 398, 262–276. 10.1016/S0140-6736(21)00536-5 34216571

[B7] Cao F. Wu K. Zhu Y. Z. Bao Z. W. (2021). Roles and mechanisms of dipeptidyl peptidase 4 inhibitors in vascular aging. Front. Endocrinol. 12, 731273. 10.3389/fendo.2021.731273 PMC8416540 34489872

[B8] Chang C. Y. Yeh Y. H. Chan Y. H. Liu J. R. Chang S. H. Lee H. F. (2017). Dipeptidyl peptidase-4 inhibitor decreases the risk of atrial fibrillation in patients with type 2 diabetes: a nationwide cohort study in Taiwan. Cardiovasc. Diabetol. 16, 159–10. 10.1186/s12933-017-0640-5 29258504 PMC5735601

[B9] Chang X. M. Xiao F. Pan Q. Wang X. X. Guo L. X. (2021). Sitagliptin attenuates endothelial dysfunction independent of its blood glucose controlling effect. Korean J. Physiol. Pharmacol. 25, 425–437. 10.4196/kjpp.2021.25.5.425 34448460 PMC8405439

[B10] Chen H. Y. Yang F. Y. Jong G. P. Liou Y. S. (2017). Antihyperglycemic drugs use and new‐onset atrial fibrillation in elderly patients. Eur. J. Clin. Investig. 47, 388–393. 10.1111/eci.12754 28369870

[B11] Chen S. Y. Kong X. Q. Zhang K. F. Luo S. Wang F. Zhang J. J. (2022). DPP4 as a potential candidate in cardiovascular disease. J. Inflamm. Res. 15, 5457–5469. 10.2147/JIR.S380285 36147690 PMC9488155

[B12] Chen Z. Yu J. Fu M. Dong R. Yang Y. Luo J. (2020). Dipeptidyl peptidase-4 inhibition improves endothelial senescence by activating AMPK/SIRT1/Nrf2 signaling pathway. Biochem. Pharmacol. 177, 113951. 10.1016/j.bcp.2020.113951 32251672

[B13] Cheng Q. Cheng J. Cordato D. Gao J. (2020). Can dipeptidyl peptidase-4 inhibitors treat cognitive disorders? Pharmacol. Ther. 212, 107559. 10.1016/j.pharmthera.2020.107559 32380197

[B14] Cornel J. H. Bakris G. L. Stevens S. R. Alvarsson M. Bax W. A. Chuang L. M. (2016). Effect of sitagliptin on kidney function and respective cardiovascular outcomes in type 2 diabetes: outcomes from TECOS. Diabetes Care 39 (12), 2304–2310. 10.2337/dc16-1415 27742728

[B15] Dai Y. Dai D. Wang X. Ding Z. Mehta J. L. (2014b). DPP-4 inhibitors repress NLRP3 inflammasome and interleukin-1beta *via* GLP-1 receptor in macrophages through protein kinase C pathway. Cardiovasc. Drugs Ther. 28, 425–432. 10.1007/s10557-014-6539-4 25022544

[B16] Dai Y. Wang X. Ding Z. Dai D. Mehta J. L. (2014a). DPP-4 inhibitors repress foam cell formation by inhibiting scavenger receptors through protein kinase C pathway. Acta Diabetol. 51, 471–478. 10.1007/s00592-013-0541-3 24363097

[B17] Davies M. J. Drexel H. Jornayvaz F. R. Pataky Z. Seferović P. M. Wanner C. (2022). Cardiovascular outcomes trials: a paradigm shift in the current management of type 2 diabetes. Cardiovasc Diabetol. 21, 144. 10.1186/s12933-022-01575-9 35927730 PMC9351217

[B18] El-Naggar A. R. Zaafar D. Elyamany M. Hassanin S. Bassyouni A. Abdel-Latif H. (2019). The role of vildagliptin in treating hypertension through modulating serum VEGF in diabetic hypertensive patients. J. Cardiovasc. Pharmacol. Ther. 24, 254–261. 10.1177/1074248418817345 30630371

[B19] Enzan N. Matsushima S. Kaku H. Tohyama T. Nagata T. Ide T. (2023). Beneficial effects of dipeptidyl peptidase-4 inhibitors on heart failure with preserved ejection fraction and diabetes. JACC Asia 3, 93–104. 10.1016/j.jacasi.2022.09.015 36873765 PMC9982295

[B20] Espeland M. A. Pratley R. E. Rosenstock J. Kadowaki T. Seino Y. Zinman B. (2021). Cardiovascular outcomes and safety with linagliptin, a dipeptidyl peptidase-4 inhibitor, compared with the sulphonylurea glimepiride in older people with type 2 diabetes: a subgroup analysis of the randomized CAROLINA trial. Diabetes Obes. Metab. 23 (2), 569–580. 10.1111/dom.14254 33185002 PMC7839453

[B21] Fadini G. P. Boscaro E. Albiero M. Menegazzo L. Frison V. De Kreutzenberg S. (2010). The oral dipeptidyl peptidase-4 inhibitor sitagliptin increases circulating endothelial progenitor cells in patients with type 2 diabetes: possible role of stromal-derived factor-1alpha. Diabetes Care 33, 1607–1609. 10.2337/dc10-0187 20357375 PMC2890368

[B22] Filion K. B. Suissa S. (2016). DPP-4 inhibitors and heart failure: some reassurance, some uncertainty. Diabetes Care 39, 735–737. 10.2337/dci15-0036 27208376

[B23] Gaggini M. Sabatino L. Suman A. F. Chatzianagnostou K. Vassalle C. (2025). Insights into the roles of GLP-1, DPP-4, and SGLT2 at the crossroads of cardiovascular, renal, and metabolic pathophysiology. Cells 14 (5), 387. 10.3390/cells14050387 40072115 PMC11898734

[B24] Guo X. Feng H. Cai L. Zheng J. Li Y. (2024). DPP-IV as a potential candidate in anti-obesity and obesity-related diseases treatment. Biomed. Pharmacother. 180, 117464. 10.1016/j.biopha.2024.117464 39326107

[B25] Gurgle H. E. White K. McAdam-Marx C. (2016). SGLT2 inhibitors or GLP-1 receptor agonists as second-line therapy in type 2 diabetes: patient selection and perspectives. Vasc. Health Risk Manag. 12, 239–249. 10.2147/VHRM.S83088 27350752 PMC4902150

[B26] Hardigan T. Abdul Y. Ergul A. (2016). Linagliptin reduces effects of ET-1 and TLR2-mediated cerebrovascular hyperreactivity in diabetes. Life Sci. 159, 90–96. 10.1016/j.lfs.2016.02.067 26898123 PMC4988948

[B27] Hwang H. J. Chung H. S. Jung T. W. Ryu J. Y. Hong H. C. Seo J. A. (2015). The dipeptidyl peptidase-IV inhibitor inhibits the expression of vascular adhesion molecules and inflammatory cytokines in HUVECs *via* Akt-and AMPK-Dependent mechanisms. Mol. Cell Endocrinol. 405, 25–34. 10.1016/j.mce.2015.01.025 25661535

[B28] Ishibashi Y. Matsui T. Maeda S. Higashimoto Y. Yamagishi S. I. (2013). Advanced glycation end products evoke endothelial cell damage by stimulating soluble dipeptidyl peptidase-4 production and its interaction with mannose 6-phosphate/insulin-like growth factor II receptor. Cardiovasc. Diabetol. 12, 125–129. 10.1186/1475-2840-12-125 23984879 PMC3765742

[B29] Jiang T. Jiang D. Zhang L. Ding M. Zhou H. (2019). Anagliptin ameliorates high glucose-induced endothelial dysfunction *via* suppression of NLRP3 inflammasome activation mediated by SIRT1. Mol. Immunol. 107, 54–60. 10.1016/j.molimm.2019.01.006 30660990

[B30] Koyama T. Tanaka A. Yoshida H. Oyama J. I. Toyoda S. Sakuma M. (2018). Comparison of the effects of linagliptin and voglibose on endothelial function in patients with type 2 diabetes and coronary artery disease: a prospective, randomized, pilot study (EFFORT). Heart Vessels 33, 958–964. 10.1007/s00380-018-1136-2 29427024

[B31] Lai S. W. Liao K. F. Lin C. L. Lin H. F. (2017). Dipeptidyl peptidase-4 inhibitors use and relative risk of ischemic cerebrovascular disease in type 2 diabetic patients in a case-control study. Front. Pharmacol. 8, 859. 10.3389/fphar.2017.00859 29213240 PMC5702655

[B32] Leopoulou M. Theofilis P. Kordalis A. Papageorgiou N. Sagris M. Oikonomou E. (2023). Diabetes mellitus and atrial fibrillation-from pathophysiology to treatment. World J. Diabetes 14 (5), 512–527. 10.4239/wjd.v14.i5.512 37273256 PMC10236990

[B33] Li D. Long Y. Yu S. Shi A. Wan J. Wen J. (2022). Research advances in cardio-cerebrovascular diseases of *ligusticum Chuanxiong* hort. Front. Pharmacol. 12, 832673. 10.3389/fphar.2021.832673 35173614 PMC8841966

[B34] Li H. Zhang J. Lin L. Xu L. (2019). Vascular protection of DPP-4 inhibitors in retinal endothelial cells in *in vitro* culture. Int. Immunopharmacol. 66, 162–168. 10.1016/j.intimp.2018.10.040 30466028

[B35] Liao L. Tang Y. Li B. Tang J. Xu H. Zhao K. (2023). Stachydrine, a potential drug for the treatment of cardiovascular system and central nervous system diseases. Biomed. Pharmacother. 161, 114489. 10.1016/j.biopha.2023.114489 36940619

[B36] Lin H. B. Li F. X. Zhang J. Y. You Z. J. Xu S. Y. Liang W. B. (2021). Cerebral-cardiac syndrome and diabetes: cardiac damage after ischemic stroke in diabetic state. Front. Immunol. 12, 737170. 10.3389/fimmu.2021.737170 34512671 PMC8430028

[B37] Liu H. Guo L. Xing J. Li P. Sang H. Hu X. (2020a). The protective role of DPP4 inhibitors in atherosclerosis. Eur. J. Pharmacol. 875, 173037. 10.1016/j.ejphar.2020.173037 32097656

[B38] Liu X. Zhang T. Zhang C. (2020b). Sitagliptin inhibits extracellular matrix accumulation and proliferation in lung fibroblasts. Med. Sci. Monit. 26, e922644. 10.12659/MSM.922644 32301442 PMC7191949

[B39] Ma S. Bai Z. Wu H. Wang W. (2019). The DPP-4 inhibitor saxagliptin ameliorates ox-LDL-induced endothelial dysfunction by regulating AP-1 and NF-κB. Eur. J. Pharmacol. 851, 186–193. 10.1016/j.ejphar.2019.01.008 30639312

[B40] Makdissi A. Ghanim H. Vora M. Green K. Abuaysheh S. Chaudhuri A. (2012). Sitagliptin exerts an antinflammatory action. J. Clin. Endocrinol. Metab. 97, 3333–3341. 10.1210/jc.2012-1544 22745245 PMC3431580

[B41] McGuire D. K. Alexander J. H. Johansen O. E. Perkovic V. Rosenstock J. Cooper M. E. (2019). Linagliptin effects on heart failure and related outcomes in individuals with type 2 diabetes mellitus at high cardiovascular and renal risk in CARMELINA. Circulation 139 (3), 351–361. 10.1161/circulationaha.118.038352 30586723

[B42] McMurray J. J. Ponikowski P. Bolli G. B. Lukashevich V. Kozlovski P. Kothny W. (2018). Effects of vildagliptin on ventricular function in patients with type 2 diabetes mellitus and heart failure: a randomized placebo-controlled trial. JACC Heart Fail 6, 8–17. 10.1016/j.jchf.2017.08.004 29032139

[B43] Meng J. Zhang W. Wang C. Xiong S. Wang Q. Li H. (2020). The dipeptidyl peptidase (DPP)-4 inhibitor trelagliptin inhibits IL-1β-induced endothelial inflammation and monocytes attachment. Int. Immunopharmacol. 89, 106996. 10.1016/j.intimp.2020.106996 33049493

[B44] Nakai M. Iwanaga Y. Sumita Y. Wada S. Hiramatsu H. Iihara K. (2022). Associations among cardiovascular and cerebrovascular diseases: analysis of the nationwide claims-based JROAD-DPC dataset. PloS One 17 (3), e0264390. 10.1371/journal.pone.0264390 35275919 PMC8916648

[B45] Nassif M. E. Kosiborod M. (2019). A review of cardiovascular outcomes trials of glucose-lowering therapies and their effects on heart failure outcomes. Am. J. Med. 132, S12–S19. 10.1016/j.amjcard.2019.10.025 31741435

[B46] Okuda Y. Omoto S. Taniura T. Shouzu A. Nomura S. (2016). Effects of teneligliptin on PDMPs and PAI-1 in patients with diabetes on hemodialysis. Int. J. Gen. Med. 9, 65–71. 10.2147/IJGM.S102070 27110135 PMC4835142

[B47] Ou H. T. Chang K. C. Li C. Y. Wu J. S. (2016). Risks of cardiovascular diseases associated with dipeptidyl peptidase-4 inhibitors and other antidiabetic drugs in patients with type 2 diabetes: a nation-wide longitudinal study. Cardiovasc. Diabetol. 15, 41–13. 10.1186/s12933-016-0350-4 26932742 PMC4774127

[B48] Ou H. T. Chang K. C. Li C. Y. Wu J. S. (2017). Comparative cardiovascular risks of dipeptidyl peptidase 4 inhibitors with other second‐and third‐line antidiabetic drugs in patients with type 2 diabetes. Br. J. Clin. Pharmacol. 83, 1556–1570. 10.1111/bcp.13241 28109184 PMC5465327

[B49] Pei J. Wang X. Pei Z. Hu X. (2023). Glycemic control, HbA1c variability, and major cardiovascular adverse outcomes in type 2 diabetes patients with elevated cardiovascular risk: insights from the ACCORD study. Cardiovasc. Diabetol. 22, 287. 10.1186/s12933-023-02026-9 37891565 PMC10612188

[B50] Pujadas G. De Nigris V. Prattichizzo F. La Sala L. Testa R. Ceriello A. (2017). The dipeptidyl peptidase-4 (DPP-4) inhibitor teneligliptin functions as antioxidant on human endothelial cells exposed to chronic hyperglycemia and metabolic high-glucose memory. Endocr 56, 509–520. 10.1007/s12020-016-1052-0 PMC5435779 27530507

[B51] Rao X. Deiuliis J. A. Mihai G. Varghese J. Xia C. Frieman M. B. (2018). Monocyte DPP4 expression in human atherosclerosis is associated with obesity and dyslipidemia. Diabetes Care 41 (1), e1–e3. 10.2337/dc17-0672 29127241 PMC5741156

[B52] Satoh-Asahara N. Sasaki Y. Wada H. Tochiya M. Iguchi A. Nakagawachi R. (2013). A dipeptidyl peptidase-4 inhibitor, sitagliptin, exerts anti-inflammatory effects in type 2 diabetic patients. Metab 62, 347–351. 10.1016/j.metabol.2012.09.004 23062489

[B53] Scheen A. J. (2018). Cardiovascular effects of new oral glucose-lowering agents: DPP-4 and SGLT-2 inhibitors. Circ. Res. 122, 1439–1459. 10.1161/CIRCRESAHA.117.311588 29748368 PMC5959222

[B54] Scirica B. M. Bhatt D. L. Braunwald E. Steg P. G. Davidson J. Hirshberg B. (2013). Saxagliptin and cardiovascular outcomes in patients with type 2 diabetes mellitus. N. Engl. J. Med. 369 (14), 1317–1326. 10.1056/NEJMoa1307684 23992601

[B55] Shih C. J. Chen H. T. Kuo S. C. Ou S. M. Chen Y. T. (2016). Cardiovascular outcomes of dipeptidyl peptidase-4 inhibitors in elderly patients with type 2 diabetes: a nationwide study. JAMDA 17, 59–64. 10.1016/j.jamda.2015.10.009 26612484

[B56] Tomovic K. Lazarevic J. Kocic G. Deljanin‐Ilic M. Anderluh M. Smelcerovic A. (2019). Mechanisms and pathways of anti‐inflammatory activity of DPP‐4 inhibitors in cardiovascular and renal protection. Med. Res. Rev. 39 (1), 404–422. 10.1002/med.21513 29806214

[B57] von Scholten B. J. Kreiner F. F. Gough S. C. von Herrath M. (2021). Current and future therapies for type 1 diabetes. Diabetologia 64, 1037–1048. 10.1007/s00125-021-05398-3 33595677 PMC8012324

[B58] Wichaiyo S. Koonyosying P. Morales N. P. (2024). Functional roles of furin in cardio-cerebrovascular diseases. ACS Pharmacol. Transl. Sci. 7 (3), 570–585. 10.1021/acsptsci.3c00325 38481703 PMC10928904

[B59] Wronkowitz N. Görgens S. W. Romacho T. Villalobos L. A. Sánchez-Ferrer C. F. Peiró C. (2014). Soluble DPP4 induces inflammation and proliferation of human smooth muscle cells *via* protease-activated receptor 2. Biochim. Biophys. Acta, Mol. Basis Dis. 1842, 1613–1621. 10.1016/j.bbadis.2014.06.004 24928308

[B60] Xie W. Song X. Liu Z. (2018). Impact of dipeptidyl-peptidase 4 inhibitors on cardiovascular diseases. Vasc. Pharmacol. 109, 17–26. 10.1016/j.vph.2018.05.010 29879463

[B61] Yang D. R. Wang M. Y. Zhang C. L. Wang Y. (2024). Endothelial dysfunction in vascular complications of diabetes: a comprehensive review of mechanisms and implications. Front. Endocrinol. 15, 1359255. 10.3389/fendo.2024.1359255 PMC11026568 38645427

[B62] Yang T. Y. Liaw Y. P. Huang J. Y. Chang H. R. Chang K. W. Ueng K. C. (2016). Association of sitagliptin with cardiovascular outcome in diabetic patients: a nationwide cohort study. Acta Diabetol. 53, 461–468. 10.1007/s00592-015-0817-x 26687195

[B63] Zannad F. Cannon C. P. Cushman W. C. Bakris G. L. Menon V. Perez A. T. (2015). Heart failure and mortality outcomes in patients with type 2 diabetes taking alogliptin *versus* placebo in EXAMINE: a multicentre, randomised, double-blind trial. Lancet 385 (9982), 2067–2076. 10.1016/s0140-6736(14)62225-x 25765696

[B64] Zelniker T. A. Wiviott S. D. Raz I. Im K. Goodrich E. L. Furtado R. H. (2019). Comparison of the effects of glucagon-like peptide receptor agonists and sodium-glucose cotransporter 2 inhibitors for prevention of major adverse cardiovascular and renal outcomes in type 2 diabetes mellitus: systematic review and meta-analysis of cardiovascular outcomes trials. Circulation 139, 2022–2031. 10.1161/CIRCULATIONAHA.118.038868 30786725

[B65] Zhang L. Chen Y. Xu J. Corpe C. P. Shtaya A. Benjamin P. (2024). Inflammation in ischemic stroke patients with type 2 diabetes–Part II: potential therapeutic targets. Adv. Neurol. 10, 1694. 10.36922/an.1694

